# Simultaneous Detection of Ricin and Abrin DNA by Real-Time PCR (qPCR)

**DOI:** 10.3390/toxins4090633

**Published:** 2012-08-31

**Authors:** Eva Felder, Ilona Mossbrugger, Mirko Lange, Roman Wölfel

**Affiliations:** Department for Medical Bio Reconnaissance and Verification, Bundeswehr Institute of Microbiology, Neuherbergstrasse 11, Munich 80937, Germany; Email: evafelder@bundeswehr.org (E.F.); ilonamossbrugger@bundeswehr.org (I.M.); mirko1lange@bundeswehr.org (M.L.)

**Keywords:** ricin, abrin, quantitative real-time PCR, simultaneous detection

## Abstract

Ricin and abrin are two of the most potent plant toxins known and may be easily obtained in high yield from the seeds using rather simple technology. As a result, both toxins are potent and available toxins for criminal or terrorist acts. However, as the production of highly purified ricin or abrin requires sophisticated equipment and knowledge, it may be more likely that crude extracts would be used by non-governmental perpetrators. Remaining plant-specific nucleic acids in these extracts allow the application of a real-time PCR (qPCR) assay for the detection and identification of abrin or ricin genomic material. Therefore, we have developed a duplex real-time PCR assays for simultaneous detection of ricin and abrin DNA based on the OmniMix HS bead PCR reagent mixture. Novel primers and hybridization probes were designed for detection on a SmartCycler instrument by using 5′-nuclease technology. The assay was thoroughly optimized and validated in terms of analytical sensitivity. Evaluation of the assay sensitivity by probit analysis demonstrated a 95% probability of detection at 3 genomes per reaction for ricin DNA and 1.2 genomes per reaction for abrin DNA. The suitability of the assays was exemplified by detection of ricin and abrin contaminations in a food matrix.

## 1. Introduction

**Figure 1:**
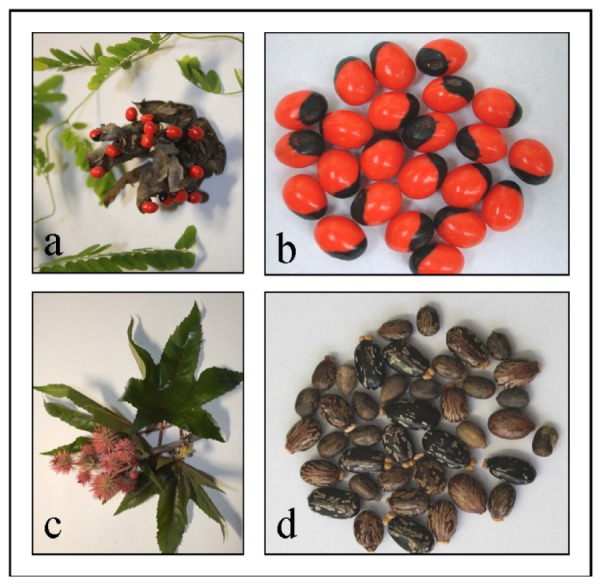
(**a**) *Abrus precatorius* plant and (**b**) jequirity seeds. (**c**) *Ricinus communis* plant and (**d**) castor seeds.

Accidental and intentional intoxications from lectins in humans and animals have been known for centuries [1]. The main causative agents hereby belong to the family of *Euphorbiaceae* (*Ricinus communis*) and *Fabaceae* (*Abrus precatorius*) [2,3]. Abrin and ricin, two of the most potent toxins known, are produced in the seeds of the jequirity plant (*Abrus precatorius*) (Figure 1 a,b) and the castor plant (*Ricinus communis*) (Figure 1 c,d) respectively. Both plants are perennial in tropical and subtropical regions of the world. Although the lethal toxicity of ricin and abrin is about 1000-fold less than that of botulinum toxin, accessibility, and ease of production in massive quantities make especially ricin a potential biological agent for terrorism. Today about 1.4 million metric tons of castor seeds are harvested annually for castor oil production [4]. Ricin as a water-soluble protein is not extracted into the castor oil, therefore industrial grade castor oil is found to be safe [5]. However, the press cake that remains after pressing and solvent extraction contains virtually all of the ricin present in the seeds (1%–5% ricin w/w), making at least ricin an easily available, inexpensive and easy to prepare toxin. The potential threat of abrin and ricin is considered significant and accordingly both toxins are classified as category B Select Agents by US Health and Human Services. 

Abrin and ricin exert toxicity through inhibition of protein synthesis, which results in cell death. Both toxins belong to the large family of ribosome-inactivating proteins and contain two disulfide-linked heterodimeric chains (A and B) with similar molecular mass (32 and 34 kDa). The A chain is an *N*-glycosidase that can irreversibly inactivate ribosomes. It cleaves a specific adenine at a highly conserved site in the 28S ribosomal RNA in the cytoplasm of eukaryotic cells, preventing binding of elongation factor 2, and thereby blocking protein synthesis [5]. The B chain is a lectin that binds to galactosyl residues of cell membranes and helps the toxin heterodimer enter target cells. Abrin and ricin can be incorporated by humans through various routes, including inhalation, injection, and ingestion [3]. Poisoning could thus also occur through ingestion of ricin- or abrin-contaminated food. The median lethal oral dose for ricin in humans has been estimated to be 1 to 20 mg/kg of body weight or 2–4 seeds on the basis of previous reports of castor bean ingestion [6], the lethal dose for children being accordingly less. Despite small molecules against the glycoprotein gpr107 from ricin, which are still under investigation [7], there are no vaccines for abrin or ricin available at the moment.

As part of an effort to provide rapid and reliable methods for the detection of various biological agents including plant toxins, a qPCR assay for simultaneous detection of ricin and abrin nucleic acid was developed.

## 2. Material and Methods

### 2.1. Sourcing of Ricin and Abrin Samples

Several cultivars of castor and jequirity seeds were obtained from two different wholesale seed traders or provided by a botanical garden (Table 1). Plant material from *Abrus pulchellus* was also included in this study as Ramos *et al.* [8] and other groups have described pulchellin, a type II ribosome-inactivating protein (RIP) toxin closely related to abrin produced by *A. pulchellus *species. Therefore it might be possible that other *Abrus* species besides *A. precatorius* express abrin or abrin-like RIP toxins. Plant material from *Cinnamomum camphora*, a genetically only distantly related lectin-producing plant, was used as a control.

Coarsely ground castor and jequirity seeds as well as crude extracts of castor seeds were processed in commercially available bread baking mixes for further analysis. The extraction procedure for the crude extract was adapted from a “terrorist cookbook” [9] to simulate an actual case sample. Bread was prepared according to the manufactures’ instructions using a household bread maker (Breadman Ultimate Bread Maker TR2200C). All contaminated labware and disposable containers were soaked in 3% hypochlorite solution for at least 24 h.

**Table 1:** Cultivars of *Ricinus communis*, *Abrus precatorius*, *Abrus pulchellus* and *Cinnamomum camphora* used in this study, including detection results using the qPCR assay described in this study.

***Species*/Cultivar**	**Source**	**Primary use**	**Detection by qPCR**	
			Ricin-specific probe	Abrin-specific probe
*R. communis* Carmencita pink	Trader 1	horticultural	**positive**	negative
*R. communis* Carmencita red	Trader 1	horticultural	**positive**	negative
*R. communis* zanzibariensis	Trader 1	horticultural	**positive**	negative
*R. communis* Green Giant	Trader 2	horticultural	**positive**	negative
*R. communis* Carmencita pink	Trader 2	horticultural	**positive**	negative
*R. communis* Carmencita red	Trader 2	horticultural	**positive**	negative
*R. communis* zanzibariensis	Trader 2	horticultural	**positive**	negative
*R. communis* Blue Giant	Trader 2	horticultural	**positive**	negative
*R. communis* Sanguineus	Trader 2	horticultural	**positive**	negative
*R. communis* Impala	Trader 2	horticultural	**positive**	negative
*R. communis* Gibsonii	Trader 2	horticultural	**positive**	negative
*R. communis* Jin2	Trader 2	agricultural	**positive**	negative
*R. communis* Fen7	Trader 2	agricultural	**positive**	negative
*R. communis* Zibo1	Trader 2	agricultural	**positive**	negative
*R. communis* Zibo 3	Trader 2	agricultural	**positive**	negative
*R. communis* Zibo 5	Trader 2	agricultural	**positive**	negative
*R. communis* Zibo 108	Trader 2	agricultural	**positive**	negative
*R. communis* CS-R6181	Trader 2	agricultural	**positive**	negative
*R. communis* Black Diamond	Trader 2	agricultural	**positive**	negative
*R. communis* Green Giant	Bot. Garden	horticultural	**positive**	negative
*A. precatorius*	Trader 1	horticultural	negative	**positive**
*A. precatorius*	Trader 2	horticultural	negative	**positive**
*A. precatorius*	Bot. Garden	horticultural	negative	**positive**
*A. pulchellus*	Bot. Garden	horticultural	negative	**positive**
*Cinnamomum camphora*	Bot. Garden	agricultural	negative	negative

### 2.2. DNA Preparation

For DNA preparation from castor seeds, one seed was sealed in a plastic bag, frozen at −80 °C and crushed manually. Material was transferred into a 15 mL tube. After addition of 3 mL distilled water and 7 mL acetone (Sigma, Munich), the tube was vigorously shaken. The suspension was sedimented for 30 min and the acetone phase was removed. Due to the high oil content of castor seeds the usage of this acetone step was necessary to produce a defatted seed pulp suitable for further processing with the DNA extraction kit. The water phase was spun down using a standard tabletop centrifuge at maximum speed and the supernatant was removed. DNA was prepared from the remaining solid phase using the DNeasy Plant Mini kit (Qiagen, Hilden) following the manufacturer’s instructions. For DNA preparation from jequirity seeds, one seed was transferred together with 100 µL PBS (Sigma, Munich) into a lysing matrix tube A (Qbiogene, Illkirch, France) with one quarter inch ceramic spheres. The seed was crushed in a Bio101 FastPrep FP120 machine (Qbiogene) for 35 s. DNA was then also prepared using the DNeasy Plant Mini kit. DNA from food matrices was extracted using the QIAamp DNA Stool Mini kit (Qiagen). Briefly, 200 mg of the food sample was mixed with ASL buffer and homogenized by vortexing. The mixture was then heated to 95 °C for 5 min. DNA was further extracted and purified as per the manufacturer’s instructions. 

### 2.3. Quantitative PCR Design

Oligonucleotide primers were designed manually based on an alignment of *Ricinus communis*, *Abrus precatorius* and other lectin-producing plant genome information. Two primer pairs were identified to be efficient for amplification of lectin-specific target sequences: RIABfor_2a (TGGGATATATGGGACAATGGAA), RIABrev_2a (AACTGCCCATTGCTGCTC), RIABfor_2r (TGGCAAATATGGGATAATGGAA) and RIABrev_2r (AGAGAGCCCATTGTTGTTCA). 

The identification and differentiation between the target sequences of ricin and abrin was enabled by two toxin-gene-specific, 5′-nuclease hybridizations probes: RIprobe_1 (6FAM-TCTAGTTTTAGCA-G-CGACATCAGGGAACAGT) and ABprobe_1 (YAK-TATCGAATGCGACAGGGCTGGCGTACA). Both probes were 3′-quenched with the BlackHole Dark Quencher 1 (BHQ1). All primers and probes were synthesized by TibMolbiol, Berlin. qPCR was performed using OmniMix HS beads (Cepheid, France) following the manufacturer’s instructions. Reaction conditions were optimized by testing variable concentrations of primers, probes and different annealing temperatures. The qPCR was run as a duplex qPCR containing 400 nM forward primers (each), 200 nM reverse primers (each) and 100 nM probes (each). A three-step PCR protocol was performed on the SmartCycler II instrument (Cepheid, France) starting with an initial denaturation step for 1 minute at 95 °C, followed by 45 cycles of 95 °C 10 s, 59 °C 20 s and 72 °C 20 s. PCR products were confirmed by agarose gel electrophoresis and GelRed staining.

### 2.4. Analysis of Assay Sensitivity

For the generation of quantifiable positive controls PCR products were cloned into the vector pCR2.1 using the TOPO-TA cloning kit (Invitrogen, Darmstadt) and analyzed by Sanger sequencing. M13 primer PCR products obtained from these positive control plasmids were used for the determination of the assay sensitivity by probit analysis: Photometrical quantified PCR products were diluted in log10 serial steps from 10^7^ to 10^0^ copies per reaction and amplified with the qPCR assay as described above. For the probit analysis, the ricin and abrin specific qPCR was run with 10, 1 and 0.5 copies DNA per reaction in 5 repeats each. Statistical analysis was done using StatGraphics Centurion XVI.I. 

### 2.5. Testing for Assay Inhibition

The presence of PCR inhibitors in the extracted DNA of all samples was assessed by spiking a known concentration of positive control DNA (described above) into a fraction of the isolated DNA from the sample. The spiked subsample was then tested by qPCR for the gene of interest and the result was compared with the test result of a non-spiked subsample.

## 3. Results

**Figure 2:**
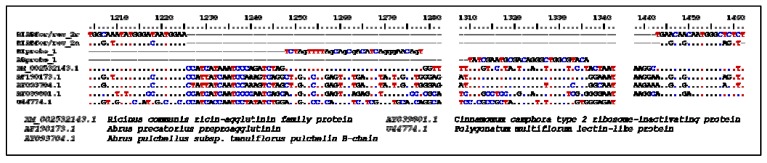
Primers and Probes used in this study; the oligonucleotide binding sites within the genome of different lectin-producing plants are shown. The scale refers to the position on the genome of *Ricinus communis* (XM_002532143.1).

The developed duplex qPCR assay for simultaneous detection of ricin and abrin DNA reliably amplified the targeted region from the genomic material of all cultivars of *R. communis* and *A. precatorius*. Results for these tests are summarized in Table 1. Ricin and abrin DNA could be distinguished by the differently labeled probes and the fluorescence signals for ricin (FAM) and abrin (Alx532) showed no crosstalk. Specific PCR products of 259 bp were confirmed by agarose gel electrophoresis in all tested positive samples (data not shown). DNA extracted from *Abrus pulchellus*, a plant producing the abrin-like toxin pulchellin, was also amplified and detected by the abrin-specific hybridization probe (see also the comparative alignment in Figure 2).

**Figure 3:**
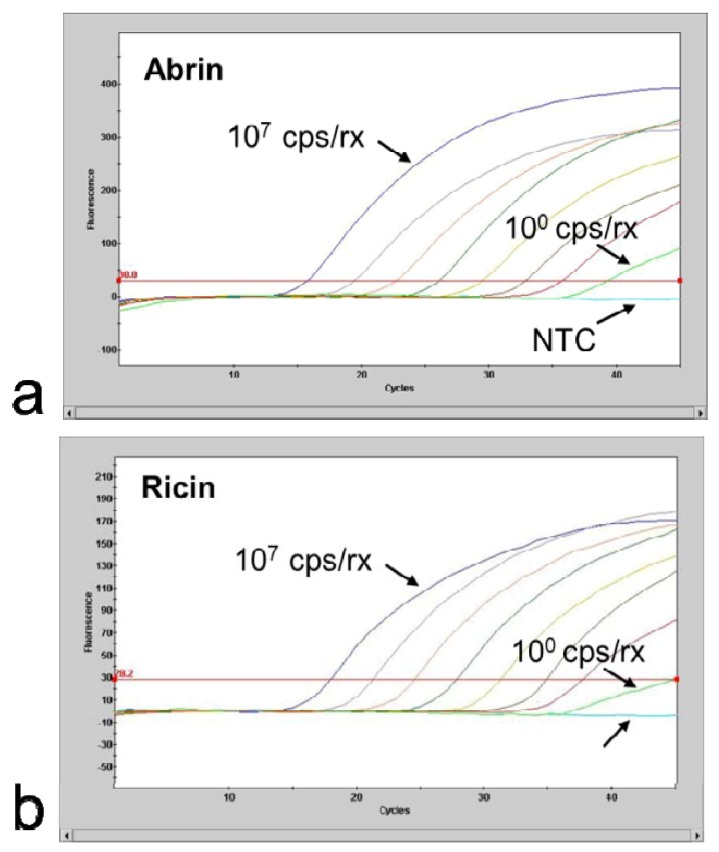
Limiting dilution series of 10^7^–10^0^ copies DNA per reaction; NTC: no template control (**a**) *A. precatorius *DNA and (**b**) *R. communis* (cultivar Carmencita pink) DNA. For further use as positive control for verification of assay performance quantified PCR products were adjusted to result in final crossing point values of 30 and dried down in a SpeedVac vacuum centrifuge for long term storage.

**Figure 4:**
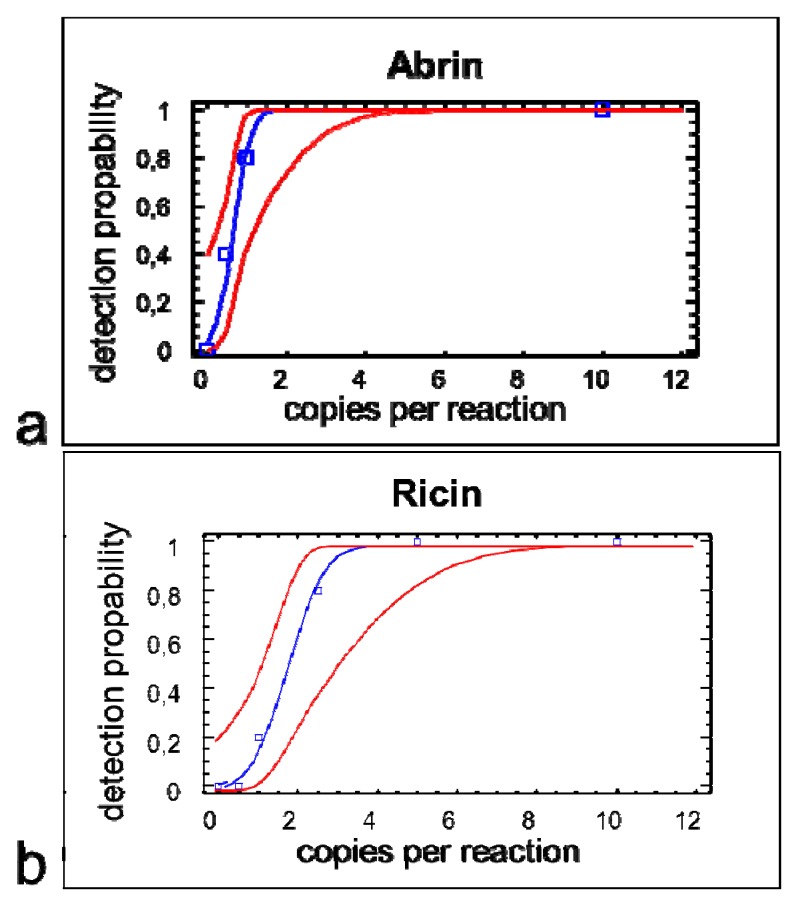
Probit analysis performed with the duplex qPCR protocol: Probability of achieving a positive test result (*y*-axis), depending on the DNA copy input number per PCR of (**a**) *A. precatorius* and (**b**) *R. communis *(cultivar Carmencita pink) DNA (*x*-axis). Upper and lower curves represent 95% confidence limits of the model.

The 95% limit-of detection (LOD) as a common technical specification indicating the concentration down to which an assay will detect the analyte with at least 95% probability, was determined by amplification of quantified ricin and abrin DNA fragments (Figure 3). For ricin 10 copies DNA per reaction could be detected in 5 of 5 qPCR runs and 1 copy DNA per reaction could be detected in 1 of 5 qPCR runs. For abrin 10 copies DNA per reaction could be detected in 5 of 5 qPCR runs, 1 copy DNA per reaction could be detected in 4 of 5 qPCR runs and 0.5 copies DNA per reaction could be detected in 3 of 5 qPCR runs. The resulting 95% LOD was 3.0 genome copies per reaction for the detection of ricin and 1.2 genome copies per reaction for the detection of abrin (Figure 4). 

Ricin and abrin DNA could be also detected in the tested food samples (Table 2). Negative inhibition of the qPCR (e.g., due to inhibitors from the food matrix) was not seen in this study. 

**Table 2:** Test results for different samples and spiked matrices.

**Sample**		**Qualitative detection by qPCR**	
		Ricin-specific probe	Abrin-specific probe
Grounded	castor seeds	**positive ^1^**	negative
	jequirity seeds	negative	**positive ^1^**
Crude extracts of	castor seeds ^2^	**positive**	negative
	jequirity seeds	negative	**positive**
Bread, containing	20 g ground castor seeds per kg	**positive**	negative
	9 g crude extract of castor seeds per kg	**positive**	negative
	8 g ground jequirity seeds per kg	negative	**positive**
Bread, control		negative	negative

^1^ all cultivars listed in Table 1 were detected; ^2^ cultivar *R. communis* “Carmencita pink”.

## 4. Discussion

The increased awareness of the potential use of abrin and ricin as biological agents for terrorism created also the need for better laboratory methods to prevent or prepare for an intentional release of abrin or ricin. Therefore several sensitive, accurate, and robust assay formats that provide critical information in a timely manner should be available. 

Among the different detection methods that are currently used, antibody-based immunoassays (e.g., ELISA) still belong to the standard technologies applied to detect and to quantify ricin and abrin in clinical or environmental samples as well as in food and feed [10,11]. An obvious disadvantage of the ELISA method is the long performance time that can take several hours, meaning that valuable time is lost, before countermeasures can be implemented. To overcome this problem, qPCR as a fast and reliable method could be of great interest as long as it is assumable, that detection of genetic material in the sample can lead to a significant conclusion for the presence of the toxin. As the production of highly purified ricin or abrin toxin is labor intensive and requires sophisticated lab equipment, it is more likely that in case of abuse as a biological agent, crude extracts, rather than purified extracts would be used by non-governmental perpetrators [12]. In such crude extracts remaining castor- and jequirity-specific nucleic acids provide appropriate surrogate analytes for the toxins themselves and allow the application of the ricin and abrin specific qPCR assay. For this reason the qPCR method described here is not only a very important method for confirmation of positive results ELISA, but can still work also in case of protein denaturation or loss, as the toxin-specific DNA is generally considered to be more stable than the toxin itself. We have validated the qPCR assay for the detection of castor and jequirity seed genome material in bread as an example for a rather complex food matrix. Potential inhibitory substances (such as polysaccharides or polyphenols) from the bread matrix could be removed through the usage of a DNA purification method which has been developed for the extraction of feces samples.

## 5. Conclusion

This study describes the use of a novel qPCR which provides a rapid and sensitive method for the detection and identification of ricin and abrin nucleic acid from a complex food matrix. Because of its high sensitivity the assay may prove useful for the investigation of deliberate or accidental castor or jequirity seed contaminations also in other kinds of substrates.

## References

[1] Flexner S. (1897). The histological changes produced by Ricin and Abrin intoxications. J. Exp. Med..

[2] Carter S., Eggli U., Albers F. (2001). Euphorbia. Dicotyledons.

[3] Dickers K.J., Bradberry S.M., Rice P., Griffiths G.D., Vale J.A. (2003). Abrin poisoning. Toxicol. Rev..

[4] Nass L.L., Pereira P.A.A., Ellis D. (2007). Biofuels in Brazil: An overview. Crop Sci..

[5] Worbs S., Kohler K., Pauly D., Avondet M.A., Schaer M., Dorner M.B., Dorner B.G. (2011). Ricinus communis intoxications in human and veterinary medicine-a summary of real cases. Toxins.

[6] Challoner K.R., McCarron M.M. (1990). Castor bean intoxication. Ann. Emerg. Med..

[7] Elling U., Taubenschmid J., Wirnsberger G., O’Malley R., Demers S.P., Vanhaelen Q., Shukalyuk A.I., Schmauss G., Schramek D., Schnuetgen F. (2011). Forward and reverse genetics through derivation of haploid mouse embryonic stem cells. Cell Stem Cell.

[8] Ramos M.V., Mota D.M., Teixeira C.R., Cavada B.S., Moreira R.A. (1998). Isolation and partial characterisation of highly toxic lectins from Abrus pulchellus seeds. Toxicon.

[9] Kinson M.H. (1988). The Poisoner’s Handbook.

[10] Poli M.A., Rivera V.R., Hewetson J.F., Merrill G.A. (1994). Detection of ricin by colorimetric and chemiluminescence ELISA. Toxicon.

[11] Griffiths G.D., Newman H., Gee D.J. (1986). Identification and quantification of ricin toxin in animal tissues using ELISA. J. Forensic Sci. Soc..

[12] Mayor S. (2003). UK doctors warned after ricin poison found in police raid. BMJ.

